# The knowledge, attitudes and practices regarding family history of hereditary diseases amongst undergraduate students at the University of the Free State

**DOI:** 10.4102/safp.v64i1.5392

**Published:** 2022-01-27

**Authors:** Lynette J. van der Merwe, Gaby Nel, Caitlin Williams, Swalica Erasmus, Riana Nel, Marize Kolver, Benine van den Heever, Gina Joubert

**Affiliations:** 1 Undergraduate Medical Programme Management, Faculty of Health Sciences, School of Clinical Medicine, University of the Free State, Bloemfontein, South Africa; 2 Department of Biostatistics, Faculty of Health Sciences, University of the Free State, Bloemfontein, South Africa

**Keywords:** hereditary diseases, family medical history, knowledge, attitudes and practices, undergraduate students, lifestyle, non-communicable diseases

## Abstract

**Background:**

Family medical history may help prevent, diagnose and treat inherited non-communicable diseases. Many people are unaware of family medical history, and medical practitioners may not realise its value when dealing with hereditary diseases. The study aimed to determine the knowledge, attitudes and practices (KAPs) regarding family medical history of hereditary diseases amongst undergraduate students at the University of the Free State, Bloemfontein.

**Methods:**

A cross-sectional study using a KAP survey was conducted. Questionnaires were distributed electronically to students for voluntary and anonymous completion.

**Results:**

There were 651 respondents (response rate 3.1%). Most respondents had good knowledge about their family history of hereditary diseases. Significantly more Health Sciences students reported knowing their family medical history. The majority knew that knowledge of hereditary diseases could improve quality of life, that they had a greater chance of being diagnosed if a family member already had the disease, and that lifestyle changes could improve health. Health Sciences students’ level of knowledge was higher than that of students from other faculties. At least 95% of students indicated that they would change lifestyle habits to prevent early onset of hereditary disease, but their practices regarding good healthcare were poor. Health Sciences students’ practices were significantly better, but less than half of all students had made lifestyle changes or had done screenings based on their family medical history.

**Conclusion:**

Although undergraduate respondents had good knowledge of family medical history and positive attitudes about screening, they did not adapt their practises. Health Sciences students’ KAPs reflected the acquisition of core competencies. Institutions could educate students on the importance of early screenings.

## Introduction

Family medical history can be a trusted source of data providing information about specific genetic disorders common in a family.^1,2^ It can assist the healthcare practitioner in determining the possibility of a hereditary genetic disorder occurring in a patient.^3^ Family medical history is helpful in preventing, diagnosing and treating common diseases, such as diabetes mellitus, cancers (e.g. breast, ovarian and colon cancer), heart disease, osteoporosis, asthma and depression.^1,4^ If patients are familiar with their family medical history, especially regarding genetic or hereditary disorders, precautionary steps may be undertaken to minimise the damage of the disorder and to possibly prevent the onset of that disorder in oneself and in future generations.^3^ Precautionary steps may include dietary and behavioural changes. For example, exercise may prevent hypertension and early testing such as a mammography may detect breast cancer.^1^

Unfortunately, many patients are unaware of their family medical history, and healthcare practitioners neglect to realise the value and relevance of such information when dealing with common disorders.^1,5^ Challenges such as time constraints and the inconvenience to collect and analyse an extensive list of family medical history information, may discourage healthcare practitioners to make use of this information and so disregard the importance thereof.^1,5^

Knowledge, attitudes and practice (KAP) surveys are regularly used to gather information to plan intervention programmes.^6^ For example, in a KAP survey regarding diabetes in type-2 diabetes patients in Iran, the study aimed to emphasise the barriers of educational programmes in Iran and show the success of improving the KAP of type-2 diabetes patients. The outcomes of the study indicated that recent informative programmes in Iran enhanced KAP levels.^7^ In rural populations in Bangladesh and Nigeria, KAP surveys were used as valuable tools providing epidemiological data regarding population health profiles.^8,9^ Most recently, KAP surveys have been used to ensure the effectiveness of disease mitigation measures and to encourage compliance in the Malaysian population with coronavirus disease 2019 (COVID-19) pandemic responses.^10^ These studies show that KAP surveys may contribute to a greater understanding of populations’ health as well as how to disseminate essential health promotion information best.

In a study examining people’s understanding of family medical history of cancer, only 54% of the 309 participating staff members at the Faculty of Medicine and Health at University of Leeds knew the type of information required to compile a family medical history. This study noted a misunderstanding and poor knowledge of family medical history and the type of information needed to build a family medical history; even in a sample of people teaching and researching medicine and health issues.^11^ Similarly, when surveying young adults online, 93% were aware of their family health history, but only 39% gathered information on it.^2^ This indicates that knowledge does not necessarily impact behaviour, even amongst healthcare trainees or professionals. It would be valuable to understand whether healthcare professionals’ attitudes regarding self-screening translates to screening of patients.

Poor KAPs regarding family medical history as well as prevention or management of hereditary diseases, could pose a potential threat to health outcomes in populations. By creating awareness and encouraging students to acquire the necessary knowledge and undertake early preventive measures, the onset of hereditary diseases may be delayed or prevented in this group. As far as could be ascertained in the literature, not many studies have been conducted to assess the KAPs regarding family history of hereditary diseases amongst undergraduate students.

The study aimed to determine the KAPs regarding family medical history of hereditary diseases amongst undergraduate students at the University of the Free State (UFS) Bloemfontein Campus. In addition, these aspects were compared between the students from the Faculty of Health Sciences and all other faculties.

## Materials and methods

### Study design, population and sampling strategy

This was a quantitative cross-sectional study. The target population included first to third-year undergraduate students registered at the UFS Bloemfontein Campus in 2018. The students of all faculties, who were willing to participate and who were 18 years of age or older at the time of the study were included. There were no exclusion criteria. Data regarding the composition of the target population obtained from the Directorate of Institutional Research and Planning (DIRAP) at the UFS (Botha J, personal communication, 2018 November 20).

### Data collection

Data were gathered by means of a self-administered, voluntary, anonymous online questionnaire, available in English. The questionnaire consisted of four sections: demographic information (four questions), knowledge of family medical history (nine questions), attitudes (eight questions) and practices regarding family medical history (eight questions). The questionnaire was designed based on the literature regarding hereditary diseases. It was made available electronically using the Evasys online survey system over a period of 12 days by means of a link sent to the students using the Blackboard learning management system. Two reminders were sent out during this period to prompt and encourage students to complete the questionnaire.

An incentive was offered in the form of a lucky draw to the value of R250.00. This was included in the approved ethics application. Students who wanted to be eligible for the lucky draw provided their email address at the end of the questionnaire. The winner of the lucky draw was selected randomly. To ensure anonymity of the winner, the information was only known to the student researchers, who handled it with discretion.

### Pilot study

A pilot study was conducted on five second-year students, who were randomly selected, from a female residence at the UFS Bloemfontein Campus. The pilot study determined the efficacy, distribution, collection and processing of the questionnaires. Relevant changes based on the feedback were made. The changes included reformulating questions to the same format with a ‘yes’/’no’ answer, and removing questions that were seen as repetitive. The data from the pilot study were not included for analysis.

### Data analysis

Data analysis was carried out by the Department of Biostatistics, Faculty of Health Sciences, UFS, using SAS version 9.4. The results were summarised by means of descriptive statistics such as frequencies, percentages (categorical variables) and means or percentiles (numerical variables). Subgroups were compared using chi-squared or Fisher’s exact tests.

The respondents’ responses to the questions on their knowledge of hereditary diseases were categorised based on their responses as excellent (> 75%) when they responded ‘yes’ to 7–8 questions, good (50% – 74%; ‘yes’ to 4–6 questions), and poor (< 50%; ‘yes’ to 1–3 questions).

### Ethical considerations

The study was approved by the Health Sciences Research Ethics Committee of the UFS [UFS-HSD2018/0361] and permission was granted by the appropriate UFS authorities. The participation was voluntary. The completion of the anonymous online questionnaire was considered as consent. All data were handled confidentially.

## Results

A total of 651 undergraduate students completed the online survey (overall response rate 3.1%). There were 421 females (65.9%; response rate 3.4%) and 218 males (34.1%; response rate 2.6%) (*n* = 639). The majority of undergraduate students at the UFS Bloemfontein Campus are female (65.9% at the time of the study) (Botha J, personal communication, 2018 November 20). This is reflected in the study results with mostly female respondents. Twelve respondents did not indicate their gender. The respondents included 227 first-year (35.4%; response rate 3.3%), 201 second-year (3.3%; response rate 3.7%), 150 third-year (23.4%; response rate 3.6%) and 64 fourth-year and beyond (9.9%; response rate 1.5%) students (*n* = 642). Nine students did not indicate their historic year of study. Students beyond the fourth year were not included in the target population, but as the response rate was very low, it was decided to include the responses from these students (*n* = 64) in the final data set.

The number of respondents from the seven faculties at the UFS are shown in Table 1. Four respondents did not indicate which faculty they were from (*n* = 647).

**TABLE 1:** Numbers of respondents from faculties at the University of the Free State (*n* = 647).

Faculty	Number of students	Percentage of sample (%)	Response rate (%)
Natural and Agricultural Sciences	129	19.9	3.1
Humanities	125	19.3	2.5
Health Sciences	117	18.1	7.3
Education	107	16.5	3.1
Economic and Management Science	105	16.2	2.8
Law	62	9.6	2.4
Theology and Religion	2	0.3	1.0

Questions that assessed the degree of familiarity about family medical history were used to determine the respondents’ knowledge. The respondents’ answers to whether they were aware of specific conditions or hereditary diseases present in their families are also shown in Table 2. Health Sciences students’ responses are indicated in a separate column and compared with students’ responses from other faculties.

**TABLE 2:** Respondents’ knowledge about family medical history of hereditary diseases.

Question		Total responses† (*n*)	Total respondents selecting ‘Yes’		Other respondents selecting ‘Yes’		Health Sciences respondents selecting ‘Yes’		
			*n*	%	*n*	%	*n*	%	*p*-value
1.	I know my family medical history	643	454	70.3	358	68.1	95	81.2	0.0049*
2.	The following are diseases that my immediate family suffer from:	454	-	-	-	-	-	-	-
	High blood pressure	-	296	65.9	330	63.5	66	56.9	0.1872
	Diabetes mellitus	-	200	44.5	213	41.2	42	36.2	0.3259
	Cancer (any form)	-	126	28.1	109	20.1	38	32.8	0.0064*
	Heart disease	-	82	18.7	63	12.1	34	29.3	< 0.0001*
	Alzheimer’s disease	-	36	8.0	30	5.8	12	11.2	0.0349*
	No hereditary diseases are present in my immediate family	-	56	12.5	62	11.9	17	14.7	0.4198
	Do not know what hereditary diseases could be present in my immediate family	-	4	0.9	39	7.5	2	1.7	0.0220*
3.	I am aware of the lifestyle changes that can improve my health regarding these diseases (with reference to the question pertaining to hereditary diseases in the participants’ immediate family)	648	522	80.6	413	78.1	108	93.1	0.0002*
4.	My chances of being diagnosed with a certain disease is higher if one of my family members have the disease	648	549	84.7	434	82.2	112	95.7	0.0002*
5.	Knowledge of hereditary (genetic) diseases can improve the quality of life	648	597	92.1	480	90.9	115	98.3	0.0069*
6.	I know of the possible medical complications I could face because of my family medical history	644	457	71.0	350	66.7	105	90.5	< 0.0001*
7.	I know of early detection methods that I should pursue (based on my family medical history)	645	288	44.7	219	41.7	68	58.1	0.0012*
8.	Hereditary diseases are curable	642	233	36.3	200	38.2	31	27.0	0.0234*
9.	I know of family members who have hereditary diseases	638	366	57.4	291	56.0	74	63.8	0.1230

* , Statistically significant difference *p* < 0.05.

† , Four students did not indicate their faculty.

Most respondents in all faculties had good knowledge about their family history of hereditary diseases, and significantly more Health Sciences students reported knowing their family medical history (*p* = 0.0049). Significant differences were seen in the knowledge of Health Science students compared to students from other faculties regarding knowledge that they had a greater chance of being diagnosed with these diseases if a family member already had the disease (82.2% of other respondents; Health Sciences 95.7%; *p* = 0.0002), awareness of lifestyle changes that could improve their health (78.1% of other respondents; Health Sciences 93.1%; *p* = 0.0002), knowledge of hereditary diseases can improve the quality of life (90.0% of other respondents; Health Sciences 98.3%; *p* = 0.0069), knowledge of possible medical complications because of family medical history (66.7% of other respondents; Health Sciences 90.5%; *p* < 0.0001), knowledge of early detection methods to pursue (41.7% of other respondents; Health Sciences 58.1%; *p* = 0.0012) and that hereditary diseases are curable (38.2% of other respondents; Health Sciences 27.0%; *p* = 0.0234). There was a statistically significant difference between Health Sciences students (29.3%) and the respondents from other faculties (12.1%) regarding knowing of family members suffering from heart disease (*p* < 0.0001), cancer (*p* = 0.0064) and Alzheimer’s disease (*p* = 0.0349). The respondents knew of family members who have hereditary diseases, for example, high blood pressure (63.5% of other respondents; 56.9% Health Sciences), diabetes mellitus (41.2% of other respondents; 36.2% Health Sciences), cancer (20.1% of other respondents; 32.8% Health Sciences), and Alzheimer’s disease (5.8% of other respondents; 11.2% Health Sciences). Significantly fewer Health Sciences students did not know what hereditary diseases were present in their immediate family (1.7%; *p* = 0.0220) compared to the respondents from other faculties (7.5%), while 11.9% of other respondents and 14.7% of Health Sciences students indicated that there were no hereditary diseases present in their immediate family.

The results regarding the respondents’ knowledge categories about hereditary diseases are shown in Table 3.

**TABLE 3:** Respondents’ knowledge categories of family history of hereditary diseases (*n* = 612).

Respondents	Categories						Knowledge		
	Poor†		Good‡		Excellent§		Median	Min–Max	Lower and upper quartiles
	*n*	%	*n*	%	*n*	%			
Health Sciences (*n* = 112)	2	1.8	40	35.7	70	62.5	7	3–8	6.0–7.5
Other faculties (*n* = 500)	79	15.8	247	49.4	174	34.8	6	1–8	4.0–7.0

† , < 50% (0–3 questions);

‡ , 50% – 74% (4–6 questions);

§ , > 75% (7–8 questions).

Health Sciences students’ level of knowledge was higher than that of students from other faculties. The majority of these students (62.5%) had answered ‘yes’ to more than 75% of the questions (median: 7), compared to 34.8% of respondents from other faculties (median: 6). This difference was statistically significant (*p* < 0.0001).

The respondents’ attitudes are shown in Table 4 in terms of their responses to statements regarding family medical history, hereditary disease and related topics. Health Sciences students’ responses are shown separately from respondents from other faculties.

**TABLE 4:** Respondents’ attitudes about family medical history of hereditary diseases.

Statement		Total responses† (*n*)	Total respondents selecting ‘Yes’		Other respondents selecting ‘Yes’		Health Sciences respondents selecting ‘Yes’		
			*n*	%	*n*	%	*n*	%	*p*-value
1.	I do care about my family medical history	646	559	86.5	449	85.4	107	91.5	0.0814
2.	It will be difficult for me to find out what diseases are/were present in my family	647	241	37.2	218	41.3	20	17.2	< 0.0001*
3.	I have thought about how my family medical history could affect my health	643	495	77.0	398	76.0	95	81.9	0.1686
4.	I would change my lifestyle habits if it would prevent me from experiencing early onset symptoms of a hereditary disease	644	617	95.8	504	96.0	110	94.8	0.6083
5.	I would not mind taking expensive, uncomfortable and time-consuming screenings if it could prolong my life	645	500	77.5	411	78.3	87	74.4	0.3571
6.	I believe that my chance of having a hereditary disease is high if a member of my immediate family already has this disease	646	519	80.3	416	79.1	100	85.5	0.1168
7.	I believe that hereditary diseases can cause death	642	428	66.7	341	65.2	84	72.4	0.1364
8.	I do not believe that everyone should get screened for hereditary diseases regardless of their family history	645	162	25.1	129	24.6	33	28.2	0.4132

* , Statistically significant difference *p* < 0.05.

† , Four students did not indicate their faculty.

The statement ‘I would change my lifestyle habits if it would prevent me from experiencing early onset symptoms of a hereditary disease’ had the highest percentage positive responses (96% of other respondents; Health Sciences 94.8%). Significantly fewer Health Sciences students (17.2%) indicated that it would be difficult to find out about diseases present in their family, compared to 41.3% of respondents from other faculties. Generally, Health Sciences students’ attitudes were more positive than those of the respondents from other faculties, except for statements about changing lifestyle habits to prevent the early onset of symptoms of a hereditary disease (as noted above) and taking uncomfortable, expensive, and time-consuming screenings if it could prolong their life (78.3% of other respondents; 74.4% Health Sciences). These differences were not significant.

The respondents’ practices, referring to the actual application of the use of information or beliefs, are shown in Table 5.

**TABLE 5:** Respondents’ practices regarding family medical history of hereditary diseases.

Statement		Total responses† (*n*)	Total respondents selecting ‘Yes’		Other respondents selecting ‘Yes’		Health Sciences respondents selecting ‘Yes’		
			*n*	%	*n*	%	*n*	%	*p*-value
1.	I do warn people about hereditary diseases	647	231	35.7	177	33.6	52	44.4	0.0265*
2.	I have already had screenings done because of family medical history knowledge	645	129	20.0	96	18.3	33	28.2	0.0155*
3.	I have already made lifestyle changes because of my family medical history knowledge	646	242	37.5	188	35.7	53	45.7	0.0436*
4.	I have conducted research on my family medical history	633	244	38.5	191	37.2	54	45.3	0.1060
5.	I have done the following check-ups in the past year:	651	-	-	-	-	-	-	-
	Blood pressure	-	384	59.0	300	57.1	79	67.5	0.0390*
	Blood sugar levels	-	305	46.9	239	45.5	63	53.9	0.1029
	Cholesterol levels	-	128	19.7	104	19.8	23	19.7	0.9703
	Full blood count	-	71	10.9	48	9.1	22	18.8	0.0024*
	Mammogram	-	21	3.2	18	3.4	2	1.7	0.5545
	Removal of malignant skin lesions	-	17	2.6	11	2.1	6	5.1	0.1016
	Colonoscopy	-	6	0.9	2	0.4	4	3.4	0.0117*
	Other	-	140	21.5	118	22.5	21	18.0	0.2823
	None	-	210	32.3	176	33.5	28	23.9	0.0439*
6.	I know of screenings that I plan on doing in my future (when I reach the recommended age)	643	405	63.0	312	59.7	92	78.6	< 0.0001*
7.	I have spoken to my family about our family medical history and the importance thereof	644	268	41.6	205	39.1	62	53.0	0.0059*
8.	I have advised people to go for screenings for hereditary diseases	633	187	29.5	141	27.4	45	38.8	0.0154*

* , Statistically significant difference *p* < 0.05.

† , Four students did not indicate their faculty.

When assessing the respondents’ practices regarding family medical history, Health Sciences students’ practices were significantly better than the respondents from other faculties in most items. This included warning people about hereditary diseases, speaking to family about medical history and its importance, advising others to go for screenings for hereditary diseases, knowing of screenings to be done in future, undergoing screenings because of knowledge of family medical history, and making lifestyle changes because of knowledge of family medical history. It is interesting to note, however, that less than half of Health Sciences students engaged in these practices except for knowing of screenings they plan on doing in the future (78.6%) or speaking to family about their family medical history (53.0%).

Most of the respondents (57.1% of other respondents; Health Sciences 67.5%; *p* = 0.0390) had undergone blood pressure check-ups in the past year, followed by blood sugar level (45.5% of other respondents; Health Sciences 53.9%), cholesterol level (19.8% of other respondents; Health Sciences 19.7%) and full blood count (9.1% of other respondents; Health Sciences 18.8%; *p* = 0.0024). Less than 10.0%, respectively, had undergone mammograms (3.4% of other respondents; Health Sciences 1.7%), removal of malignant skin lesions (2.1% of other respondents; Health Sciences 5.1%) or a colonoscopy (0.4% of other respondents; Health Sciences 3.4%). About one-third of the respondents from other faculties (33.5%) had not undergone any check-ups in the past year, compared to significantly fewer Health Sciences students (23.9%).

## Discussion

In this study, the KAPs of undergraduate students at the UFS regarding family history of hereditary diseases revealed that the respondents’ knowledge about family medical history, hereditary diseases and prevention was generally good. Most respondents in this study reported knowing their family medical history, and the conditions most frequently reported included high blood pressure, diabetes mellitus, cancer and heart disease. Very few respondents reported no history of hereditary disease in their immediate family.

Overall, Health Sciences students’ knowledge was better than that of students from other faculties. Significant differences were seen in the responses to the questions regarding knowledge of family medical history, lifestyle changes that can improve health, chances of being diagnosed with a certain disease if it is present in a family member, possible medical complications because of family medical history, and knowledge about immediate family members suffering from heart disease or early detection methods that they could pursue. This is gratifying, considering that studies conducted amongst healthcare staff reported that only 54% had enough knowledge about their family medical history related to cancer.^11^

When categorising knowledge responses, Health Sciences students’ levels of knowledge were significantly higher than that of respondents from other faculties. This finding may be expected based on the curriculum content of Health Sciences programmes, but also indicates that students may have already assimilated the competencies of ‘Health Advocate’, ‘Communicator’ and ‘Scholar’ as required by the Health Professions Council of South Africa for the training of undergraduate healthcare professionals.^12^ The World Health Organization (WHO) reported that the mortality from chronic disease will increase by 17% from 2005 to 2015, and that hereditary factors are common non-modifiable risk factors for chronic disease.^13^ Knowledge about family medical history and subsequent behavioural changes are therefore important in the prevention of chronic disease.

Although most respondents knew that lifestyle changes could prevent the onset of certain diseases (78.1% of other respondents; 93.1% Health Sciences students), only 41.7% of respondents from other faculties knew about early-detection methods for hereditary diseases. This concurs with literature on breast self-examination as an early detection method for breast cancer in resource-limited settings carried out amongst secondary and tertiary students in Ghana. The authors concluded that knowledge was low on breast self-examination, and it is comparable with the findings from studies conducted in Cameroon and Uganda.^14^ In contrast, more than half of Health Sciences students knew about these early detection methods (58.1%). This finding is also unsurprising in the light of the curriculum content of Health Sciences programmes and provides data on knowledge amongst Health Sciences students who are not readily available in the literature.

The respondents’ attitudes regarding family medical history were generally positive. Most of them indicated that it is very important to undergo screening for hereditary diseases, and that they have thought of how family diseases could affect them. Once again, Health Sciences students’ responses were slightly better than those of the respondents from other faculties regarding their attitudes. The only significant difference seen was amongst Health Sciences students with only 17.2% indicating that it would be difficult to discover diseases present in their families, thereby supporting their achievement of the core attribute of ‘Communicator’ required of healthcare professionals.^12^

The respondents’ attitudes indicated that they would go to great lengths to improve their lifestyle if it could prolong life, even if it meant having uncomfortable or expensive tests carried out. In a study on colorectal screenings, however, focus group findings indicated that there might be various complex barriers to patients undergoing screening tests, which should be considered when attempting to influence screening practices.^15^

Although the respondents in this study had good knowledge and positive attitudes towards their family medical history, it did not reflect in their practices. More than half of the respondents had carried out certain medical check-ups, yet they reported that they have not specifically undergone testing for hereditary diseases. This concurs with studies on knowledge and the prediction of behaviours regarding diabetes mellitus.^8^ Similarly, good knowledge and attitudes did not lead to the necessary practices that could improve health regarding chronic disease (including hypertension and diabetes mellitus) amongst rural populations.^8^

In general, Health Sciences students’ practices were significantly better than those of the respondents from other faculties, except for researching family medical history and certain medical check-ups. Although the majority of Health Sciences students indicated that they knew of screenings they plan on doing in future and that they had spoken to their family about medical history, less than half of Health Sciences students had made lifestyle changes because of knowledge of their family medical history or had advised people to go for screenings for hereditary diseases. While the respondents had mostly undergone blood pressure and blood glucose level checks, almost a quarter of Health Sciences students (23.9%) and a third of the respondents from other faculties (33.5%) had not had any check-ups in the past year. This may indicate a gap between the respondents’ knowledge and attitudes translating into practices. This was also seen amongst medical students in the United States, whose practices regarding sun-protection or skin self-examination were less than optimal.^16^ In a study on the knowledge and attitudes of breast cancer and practices regarding breast self-examination amongst female university students in Jordan, the implementation of a 1-day educational programme led to significant improvements in KAPs.^17^ These findings support increasing awareness and education regarding family medical history.

In this study, Health Sciences students’ KAPs regarding family medical history were better than those of the respondents from other faculties. This illustrates that they are achieving the core competencies ‘Health Advocate’, ‘Communicator’, and ‘Scholar’ as adopted by the Health Professions Council of South Africa from the CanMEDs competency framework in the training of healthcare professionals.^12^ Educational programmes should actively address the achievement of this competency to ensure that future healthcare professionals effectively educate, counsel, diagnose and manage patients.

The overall response rate in this study was low (3.1%), thereby limiting the generalisability of the results. The participating students may have been those most knowledgeable about or interested in the topic. The study respondents were mostly female (65.9%), representing the overall gender distribution of undergraduate students at the UFS Bloemfontein Campus (65.9%) (Botha J, personal communication, 2018 November 20). The response rate for females (3.4%) was higher than that of males (2.6%). The poorer response rate amongst male students is supported by WHO reports regarding male health behaviours.^18^ In our study, the highest response rate was from students in the Faculty of Health Sciences (7.3%). However, it is still a very poor response rate amongst students who could be regarded as the healthcare models for future generations.^16^ The response rates did not improve with the years of study, possibly suggesting that age and maturity may not play a role in the health behaviours of student populations. Previous studies regarding the health behaviours amongst students did not refer to response rates.^2,16,19^

Creating awareness regarding family medical history, encouraging lifestyle interventions to prevent chronic diseases, for example, hypertension and diabetes mellitus, and screening for these are essential, even amongst student populations.^2^ The findings from this study have highlighted that, although undergraduate students seem to have good knowledge and attitudes regarding family medical history, their practices poorly reflect this. Moreover, very low response rates in this KAP survey emphasised a lack of awareness or interest amongst this student population.

### Study limitations

This study had several limitations. The low response rate in this study limits the generalisation of the findings. Self-reported data could be misleading, as the respondents may not have understood all the health-related terminology used in the questions despite definitions being provided. The KAP surveys are also limited in the sense that they provide only an overview of a topic and lack in-depth information. Future studies amongst undergraduate students may explore alternative options of questionnaire distribution or alternative methodologies to encourage participation.^20^

## Conclusion and recommendations

The good knowledge about hereditary diseases and the positive attitudes towards family medical history seen in this study, especially amongst Health Sciences students, was poorly reflected by the performance of practices regarding early preventative measures, such as screenings. Undergraduate students do not practise good self-care for hereditary diseases in their family. Health Sciences students’ KAPs about family medical history reflected the acquisition of the core competencies ‘Health Advocate’, ‘Communicator’, and ‘Scholar’ as required by the Health Professions Council of South Africa.

Further studies in undergraduate student populations at other tertiary institutions should be encouraged to increase awareness and improve health in future generations. This includes knowledge about family medical history that may lead to lifestyle interventions, early screening and detection and possible prevention of the onset of diseases or optimal disease management. Interventions and awareness days could be initiated in secondary/tertiary educational institutions, as well as in the general community to inform and educate people on hereditary diseases and the importance of early screenings. The value of gathering family medical history could be encouraged by educating healthcare practitioners and the public about the effectiveness and the significance that it may hold.
